# A Genome-Wide Screen in Yeast Identifies Specific Oxidative Stress Genes Required for the Maintenance of Sub-Cellular Redox Homeostasis

**DOI:** 10.1371/journal.pone.0044278

**Published:** 2012-09-06

**Authors:** Anita Ayer, Sina Fellermeier, Christopher Fife, Simone S. Li, Gertien Smits, Andreas J. Meyer, Ian W. Dawes, Gabriel G. Perrone

**Affiliations:** 1 University of New South Wales, Sydney, Australia; 2 Swammerdam Institute for Life Sciences, University of Amsterdam, Amsterdam, The Netherlands; 3 INRES- Chemical Signalling, University of Bonn, Bonn, Germany; 4 University of Western Sydney, Penrith, Australia; University of Kent, United Kingdom

## Abstract

Maintenance of an optimal redox environment is critical for appropriate functioning of cellular processes and cell survival. Despite the importance of maintaining redox homeostasis, it is not clear how the optimal redox potential is sensed and set, and the processes that impact redox on a cellular/organellar level are poorly understood. The genetic bases of cellular redox homeostasis were investigated using a green fluorescent protein (GFP) based redox probe, roGFP2 and a pH sensitive GFP-based probe, pHluorin. The use of roGFP2, in conjunction with pHluorin, enabled determination of pH-adjusted sub-cellular redox potential in a non-invasive and real-time manner. A genome-wide screen using both the non-essential and essential gene collections was carried out in *Saccharomyces cerevisiae* using cytosolic-roGFP2 to identify factors essential for maintenance of cytosolic redox state under steady-state conditions. 102 genes of diverse function were identified that are required for maintenance of cytosolic redox state. Mutations in these genes led to shifts in the half-cell glutathione redox potential by 75-10 mV. Interestingly, some specific oxidative stress-response processes were identified as over-represented in the data set. Further investigation of the role of oxidative stress-responsive systems in sub-cellular redox homeostasis was conducted using roGFP2 constructs targeted to the mitochondrial matrix and peroxisome and *E*_GSH_ was measured in cells in exponential and stationary phase. Analyses allowed for the identification of key redox systems on a sub-cellular level and the identification of novel genes involved in the regulation of cellular redox homeostasis.

## Introduction

The maintenance of an optimal redox environment is critical to the functioning of many biological processes. The majority of intracellular functions require a strongly reducing environment, which maintains key sulfhydryl groups in a reduced and active form and other key elements of proteins and enzymes to remain functional. In eukaryotic cells, highly reducing environments are found in compartments including the cytosol, mitochondrial matrix and peroxisome. Conversely, optimal oxidative protein folding and maturation requires disulfide bond formation and a more oxidative redox environment, found in the endoplasmic reticulum [1], [2] and mitochondrial inter-membrane space [3]–[5]. Maintenance of appropriate cellular and sub-cellular redox environments is crucial since alterations in redox can adversely affect biological processes such as signal transduction [6], protein structure an function, DNA and RNA synthesis [7], enzyme activation and regulation of the cell cycle [8], [9]. Moreover, altered cellular redox homeostasis has been associated with neurodegenerative disorders including Alzheimer’s and Parkinson’s diseases [10] and metabolic syndromes such as diabetes [11].

Despite the importance of redox homeostasis for fundamental cell biology and many disease states, our current understanding has been limited by available assay methods relying on whole-cell lysate analysis and the use of altered resistance to oxidants as a phenotypic indicator of redox imbalance or dysfunction. While the thioredoxin (TrxSS/Trx(SH)_2_) system, glutathione system (GSSG/2GSH), the NADP^+^/NADPH couple, protein thiols and antioxidant defence systems have all been observed to impact on redox homeostasis on a cell-wide level [9], [12], [13], the exact genetic mechanisms in place for the maintenance of redox homeostasis are still unknown. Studying how cells regulate redox state on a sub-cellular level could lead to a better understanding of how aberrant redox biology may impact on cellular health in a variety of settings.

In order to gain a better insight into the genetic organization underpinning cellular redox homeostasis, a genome-wide screen of cytosolic redox state was carried out using the *Saccharomyces cerevisiae* tetO_7_ promoter replacement collection (essential genes) [14] and the homozygous diploid non-essential gene deletion collection [15]. Due to its relatively high concentration (in the millimolar range) and low half-cell reduction potential at pH 7 (−240 mV), the glutathione couple is considered the primary cellular redox buffer and its redox state state (*E*_GSH_) is used as an indicator of the state of both cellular and/or sub-cellular redox environment. Cytosolic redox potential (*E*_GSH_) was systematically analyzed to identify genes affecting cytosolic redox homeostasis. Redox measurements were determined using the roGFP2 probe, which predominantly equilibrates with the GSSG/2GSH redox couple [16], [17] and acts as an *in vivo* sensor of this couple. 102 genes were identified as essential for maintenance of cytosolic redox state, the majority of which were previously not known to be important for redox homeostasis. Specific oxidative stress genes were over-represented in the genes identified and by taking advantage of the ability of roGFP2 to be targeted to individual sub-cellular compartments, a targeted screen of 42 mutants affected in oxidative stress pathways in the cytosol, mitochondrial matrix and peroxisome was conducted to better understand the systems required to maintain redox homeostasis on a sub-cellular level.

## Results

### Verification of Redox-responsiveness of roGFP2-based Probes

Probes used to measure the redox state of the GSSG/2GSH couple based on the roGFP2 probe that were targeted to the cytosol and mitochondrial matrix were generated and verified as described in [18] and localization of peroxisome-targeted roGFP2 was analyzed by confocal microscopy in this study (Figure S1). In yeast cells expressing roGFP2-SKL a punctate fluorescent pattern consistent with peroxisomal localization was observed. Additionally, we also expressed roGFP2-SKL in *pex5* cells, which are defective in the import of peroxisomal proteins with a peroxisomal targeting signal 1 (PTS1) such as -SKL. In *pex5* cells, a cytosolic fluorescent pattern was observed indicative that roGFP2-SKL construct was correctly targeted and is imported via the PTS1-mediated import pathway into the peroxisome.

The roGFP2 probes response to redox state has been calibrated *in vitro* (18). Additionally, the responsiveness to *in vivo* redox state changes. To verify that roGFP2 in organelle-targeted plasmids was redox responsive *in vivo*, wild-type cells expressing organelle-targeted roGFP2 were grown to exponential phase in SC_URA_ and treated with various concentrations of dithiothreitol, a strong thiol-containing reductant or diamide a known GSH oxidizing agent. Wild-type cells treated with various concentrations of DTT, resulted in only a small change in *E*_GSH_ indicating that in the cytosol, peroxisome and mitochondrial matrix the roGFP2 probe was almost entirely in a fully reduced state (Figure S2A). Conversely, diamide-treated cells displayed progressively more oxidized *E*_GSH_ values that reached a plateau when the reporter was at its oxidized limit (Figure S2A). This verified that the organelle-targeted roGFP2 probes were able to respond dynamically from its most reduced to most oxidized state to intracellular redox state changes. To verify that changes observed via flow cytometry were due to changes in the redox-state of the roGFP2 protein *in vivo,* redox western blots were carried out to determine the redox status of the roGFP2 probe in untreated cells and cells treated with diamide (10 mM; 20 min) or DTT (10 mM; 20 min). Proteins were harvested by trichloroactetic acid precipitation and protein concentration determined by a detergent-compatible Bradford protein assay (Figure S2B). The western blots indicate that in the cytosol, mitochondrial matrix and peroxisome the roGFP2 probe was almost completely reduced in untreated conditions. The roGFP2 protein extracted from untreated cells had mobility identical to the protein extracted from cells treated with 10 mM DTT. Treatment with diamide resulted in a shift in the protein migration corresponding to that of the oxidized roGFP2 protein. Conversely, wild-type GFP (non-redox sensitive GFP) did not respond to treatment with either diamide or DTT. Together these data indicate that the organelle-targeted roGFP2 probes can sense changes in the intracellular redox environment and are responsive to changes to redox changes.

### High Throughput Screen of the Yeast Essential and Non-essential Deletion Libraries Identifies 102 Genes Required for Cytosolic Redox State Maintenance under Steady-state Conditions

A genome-wide screen was carried out to identify mutants affected in cytosolic redox environment using the redox-sensitive green fluorescent protein (roGFP2). The collection of 4,800 homozygous diploid mutants, each deleted for one non-essential gene [15] was transformed with the roGFP2 construct. Cells were grown to exponential phase at 25°C in SC_URA_ in 96-well plate format and the ratio of the intensity of emission after excitation at 405 nm and 488 nm (R_405/488_) was determined for each mutant. The primary round of screening was conducted using a 96-well plate method and a fluorescent spectrophotometer to estimate the emission ratio. All potential mutants showing a shift in R_405/488_ and any decrease in emission after excitation at 488 nm and/or any increase in emission after excitation at 405 nm were subsequently rescreened using flow cytometry, which was found to be more sensitive. An increase in the R_405/488_ indicated a shift in the cytosolic redox state towards a more oxidized environment. Mutants which displayed statistically different R_405/488_ values compared to wild type using a p-value of 0.001 were then identified. To rule out false positives, mutants were treated with 10 mM dithiothreitol (DTT) as a strong reductant. If the change in R_405/488_ was not redox dependent, for example due to the accumulation of autofluorescent metabolites or cell-wall components, treatment with DTT would not significantly alter the R_405/488_ in a pronounced way. However, if the change in R_405/488_ was redox-dependent, DTT treatment would restore the R_405/488_ to that of the wild type. After this stringent testing process, 89 mutants with a significantly higher R_405/488_ compared to wild type were identified. To allow for pH-corrected *E*_GSH_ values, the cytosolic pH of the wild type was determined to be pH 7.5 using the cytosolic pHluorin probe. This value was used for the determination of cytosolic redox potential (*E*_GSH_) and the cytosolic pH of identified mutants was verified to be similar to the wild type. Table 1 lists the top 15 mutants showing a significantly more oxidized cytosol than the wild type with the full list of mutants identified given in Dataset S1.

**Table 1:** List of non-essential *S. cerevisiae* genes that when deleted significantly affect cytosolic redox state.

Mutant	Function	*E*_GSH_±SD (−mV)
Wild type	–	349±3
*glr1*	Cytosolic/mitochondrial glutathione reductase	275±4
*yap1*	Oxidative stress responsive transcription factor	319±4
*skn7*	Oxidative stress responsive transcription factor	320±5
*dog2*	2-deoxyglucose-6-phosphate phosphatase	325±4
*dig1*	MAP kinase-responsive inhibitor of the Ste12p	327±6
*dgr2*	Protein of unknown function	330±4
*dit1*	Sporulation-specific enzyme	330±3
*tkl1*	Transketolase	330±4
*ipk1*	Inositol 1,3,4,5,6-pentakisphosphate 2-kinase	332±3
*ysp1*	Mitochondrial protein	332±4
*nvj1*	Nuclear envelope protein	332±3
*rpl26b*	Component of the large (60S) ribosomalsubunit	332±6
*inp2*	Peroxisome-specific receptor	332±6
*yju3*	Monoglyceride lipase	332±5
*ubi4*	Ubiquitin	332±4

Mutants are listed from most to least oxidized.

The screen of essential gene mutations was carried out using the tetO_7_ promoter replacement (tetO_7_) collection [14], which contains ∼ 800 mutants, each mutant has a Kan^R^-tetO_7_-TATA_CYC1_ cassette integrated into the promoter of an essential gene and the tetracycline transactivator (tTA) integrated at the *URA3* locus to form a “tet-off” system. Addition of doxycycline to the medium represses expression of genes under the control of the tetO_7_-_CYC1_ promoter. Mutants of the tetO_7_ collection were separately transformed with the cytosolic roGFP2 construct (leucine-based selection) and grown at 25°C in SC_LEU_ in 96-well plate format in the presence of doxycycline. The ratio of the intensity of emission after excitation at 405 nm and 488 nm (R_405/488_) was determined for each mutant grown after 24 hours and R_405/488_ calculated. As with the non-essential homozygous diploid collection, mutants from the tetO_7_ collection having an R_405/488_ significantly greater than the wild type were treated with DTT to identify redox-dependent changes and cytosolic pH was measured. 13 genes were identified with a significantly higher R_405/488_ compared to the wild type and the cytosolic redox potential (*E*_GSH_) was calculated (Table 2).

**Table 2:** Mutants from the tetO_7_ promoter replacement collection that exhibited a significantly more oxidized cytosol than wild type after treatment with doxycycline.

Gene	Function	*E*_GSH_±SD (−mV)
Wild type		322
tet-O_7_-*STT4*	Phosphotidylinositol-4-kinase	272±6
tet-O_7_-*SRP14*	Signal recognition particle complex subunit	282±16
tet-O_7_-*yor146w*	Dubious opening reading frame	284±6
tet-O_7_-*CDC34*	Ubiquitin-conjugating enzyme	291±7
tet-O_7_-*YPT1*	Rab family GTPase	298±14
tet-O_7_-*SPC98*	Component of the microtubule-nucleating Tub4p complex	298±3
tet-O_7_-*SAH1*	S-adenosyl-L-homocysteine hydrolase	298±6
tet-O_7_-*SEC53*	Phosphomannomutase	299±5
tet-O_7_-*RIB5*	Riboflavin synthase	299±6
tet-O_7_-*TRR1*	Cytoplasmic thioredoxin reductase	300±4
tet-O_7_-*GPI16*	Transmembrane protein subunit of the glycosylphosphatidylinositol transamidase complex	307±6
tet-O_7_-*NUP159*	Nucleoporin subunit of the nuclear pore complex	310±10
tet-O_7_-*NAB3*	Single-stranded RNA binding protein	310±3

### Functional Analysis of Genes Identified

**Figure 1:**
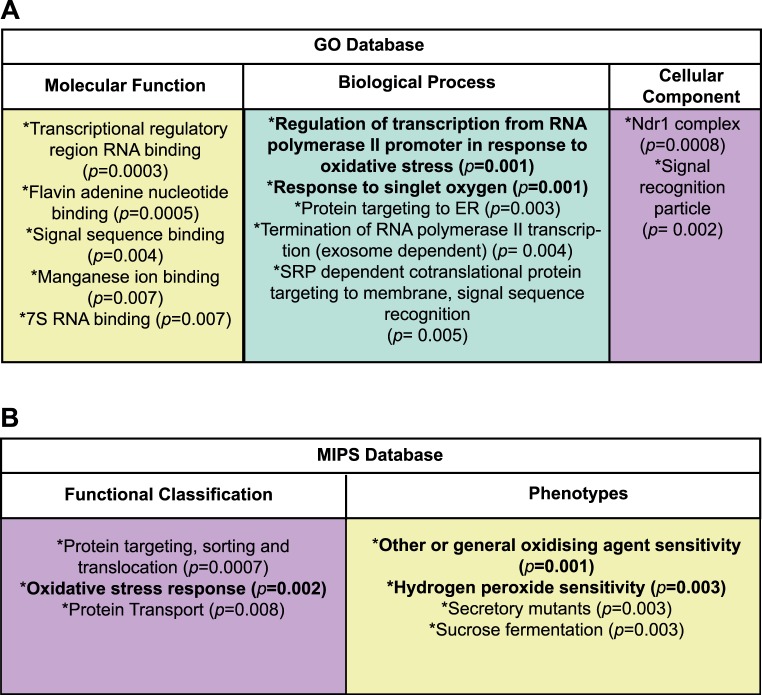
Characterization of mutants displaying a significantly more oxidized cytosol relative to wild type. All mutants that exhibited a significantly more oxidized cytosol compared to wild type were analyzed for enriched terms using the Gene Ontology (GO) and Munich Information Center for Protein Sequences (MIPS) databases via FunSpec [18]. *p*-values (Boneferroni corrected) represent the probability that the intersection of a given list within any functional category occurs by chance. The terms identified that are related to oxidative stress are highlighted in bold.

The mutants identified in this screen were analyzed to identify significantly enriched (*p*<0.01) cellular processes and functions using the Gene Ontology and Munich Information Center for Protein Sequences (MIPS) databases via FunSpec [19] as described in the Materials and Methods. Importantly, Funspec analysis showed no enrichment for slow growing mutants, indicating that altered growth alone does not affect cytosolic redox state. As seen in Figure 1, the significantly enriched terms generated from these databases represent a range of cellular processes and functions with the two databases categorizing the data in appreciably different ways. From this analysis, genes involved in endoplasmic reticulum protein transport and translocation including components of the signal recognition particle (SRP) complex were enriched in the data set. While we could not study the redox state of the endoplasmic reticulum since it is too oxidizing to be measured by roGFP2, it is evident that defects in endoplasmic reticulum protein translocation affect cytosolic redox state. Mutations in SRP and downstream effects could be through the increased cytosolic accumulation of secretory protein precursor and chaperones or through the accumulation of misfolded proteins in the endoplasmic reticulum (as suggested by [20]) leading to oxidative stress through rounds of operation of the protein folding machinery.

Many of the genes identified possibly represent new functions important in redox homeostasis. *DOG2,* which encodes 2-deoxyglucose-6-phosphatase, is induced by oxidative and osmotic stress [20] and the results point to it having a significant role in regulating redox homeostasis, more so than its current functional description indicates. Additionally, the identification of genes that have near identical duplicates (for example *HTA1* and *RPL26*) or part of a greater protein complex (*LEA1*; required for U2 snRNP) suggest that there are a sub-set of genes that moonlight as redox sensors. There are two genes encoding *RPL26* in *S. cerevisiae*, *RPL26A* and *RPL26B* which are nearly identical to *HTA2,* yet only mutation to *RPL26B* and *HTA1* caused changes in cytosolic redox state. The fact that only one mutation has such a profound effect on redox homeostasis indicates that genes such as *RPL26B* and *HTA1* may have a specific function in redox homeostasis aside from their main function. Alternatively, the gene identified may be expressed more highly than its homologue leading to its deletion having a greater effect on redox homeostasis. In the same vein, the identification of only one gene in a protein complex such as *LEA1* (component of the U2 snRNP complex) *or PXA1* (subunit of the peroxisomal ATP-binding cassette transporter complex; Pxa1p-Pxa2p), may indicate a specialized role for these genes in redox homeostasis.

While functional categorization of gene lists using GO terms is especially useful when working with large amounts of data, often such terms are not sufficiently fine or descriptive to identify functional groups or allocate genes whose products have pleiotropic effects or are less studied, into established groups. Closer inspection of the data revealed that processes affecting cytosolic redox homeostasis such as mitochondrial function (*YPS1, NDE1, MRPL25, MRPS16, MRPL35, MRPL24, ICP55, MNE1, MSF1, MRPL40, COQ10, MSW1, TIM8, AIM38*) and the ubiquitin system **(** *UBI4, UIP4, UBP13, CDC34* **)** were not detected by either the GO or MIPS databases. These represent new pathways somehow involved in redox homeostasis. Respiratory-incompetent cells have increased levels of GSSG in stationary phase relative to exponential phase and mitochondrial function is required for resistance to oxidative stress [21]. Ccp1p is also involved in oxidative stress signaling leading to activation of the Skn7p transcription factor [22]. This could explain the larger shift in matrix redox state observed in the *ccp1* mutant compared to the *skn7* mutant. Deletion of *CCP1* may cause increased levels of hydrogen peroxide to accumulate and decreased activation of Skn7p doubly affecting matrix redox state. Taken together, it is apparent that there is a connection between mitochondrial function and redox homeostasis. The link between mitochondrial function and redox homeostasis may be due to several reasons. Many mitochondrial processes are integral to the generation of redox-active species such as NADH and NADPH, they involve redox-dependent electron transfer reactions and produce reactive oxygen species. Consequently, the redox-association of many mitochondrial functions may indicate they have a potential role in the setting, sensing or maintenance of the cellular redox environment.

Four mutants (*ubi4, uip4, ubp13* and *cdc34*-tetO7) involved in ubiquitin-dependent protein degradation were identified as having a more oxidized cytosol than the wild type. Moreover, decreased expression of *CDC34*, an essential gene which encodes a ubiquitin-conjugating enzyme [23], led to a more oxidized cytosol. Mutants of the ubiquitin system were previously identified in a screen for mutants that over-excrete glutathione [24] and *CDC34* was identified as a high-copy suppressor of the poor growth phenotype of *gsh2* cells, which lack the second step of glutathione synthesis [25]). *CDC34* was shown to activate *GSH1* (encoding γ-glutamylcysteine synthetase) expression by modulating the activity of Met4p, the transcriptional activator of the sulphur assimilation pathway [25], providing a link between the GSH and ubiquitin systems. Given that overexpression of *CDC34* increases GSH levels and repression of *CDC34* leads to a more oxidized cytosolic redox state, tight control of Cdc34p is required for optimal intracellular glutathione concentrations, and alterations in the activity of Cdc43p would affect glutathione homeostasis. Disruption to the ubiquitin system and to protein degradation processes on a broad level not only via Cdc34p, may affect *GSH1* gene expression and regulation of the glutathione couple and redox state.

### Specific Oxidative Stress Responsive Genes are Essential for Cytosolic Redox State Homeostasis

Interestingly, both databases highlighted the enrichment of genes involved in oxidative stress and response to reactive oxygen species as shown in bold in Figure 1. Some oxidative-stress responsive genes identified showed the most extensive shift in *E*_GSH_ compared to the wild type (Δ*E*_GSH_), with the *glr1*, *yap1*, *skn7* and *tkl1* mutants being in the top 10 mutants with the largest Δ*E*_GSH_ values. Genes involved in the detoxification of reactive oxygen species have conventionally been regarded as response systems rather than maintenance systems. However the results of the screen indicate that some key oxidative stress responsive systems serve a dual role. This challenges the paradigm that maintenance and response systems are distinct elements in redox regulation and supports the view that some of the systems that are needed in the response to counteract deleterious oxidative changes are required to maintain redox homeostasis under steady state conditions. Clearly, some genes identified in this screen will indirectly affect the redox environment rather than being direct modulators of cytosolic redox state. For instance it is possible that mutants may have increased levels of oxidative stress, which could affect the roGFP2 readout rather than directly affecting the glutathione couple. However, delineating between primary and secondary redox effects is complex and not the focus of this present study. Additionally, it is interesting that ∼10% of the genes identified in this screen have no known or putative function. These now can be seen to have some involvement, directly or indirectly in modulating cytosolic redox state.

### Targeted Cross-compartmental Screen to Understand the Role of Oxidative-stress Systems in Redox Homeostasis

From the mutants identified in the screen, only five (*glr1*, *yap1*, *skn7, tkl1* and *trr1*; [12], [26]) have been previously reported to be involved in redox homeostasis. Some genes previously identified as affecting cellular redox state were not identified through this screen possibly due to the differences in methods used to estimate *E*_GSH_ and redundancy of some known antioxidant functions. Given that genes involved in antioxidant function and resistance to oxidative stress were identified as important to cytosolic redox maintenance through the genome-wide screen, we then looked more closely at known pathways involved in antioxidant function and oxidative stress responses to understand better their role in setting, sensing and maintenance of redox state. Moreover, these analyses were carried out on a compartmental level (in the cytosol, mitochondrial matrix and peroxisome) to understand the role of these functions in these organelles and the nature of systems maintaining redox state at a sub-cellular level.

For this study, cells transformed with compartment-targeted roGFP2 constructs were grown in SC_URA_ to exponential (A_600_ = ∼0.5) and stationary phase (A_600_ = 5–6) and analyzed via flow cytometry. *E*_GSH_ was measured in the cytosol, mitochondrial matrix and peroxisome of 42 mutants affected in the transcriptional response to oxidative stress, NADPH generation, the thioredoxin and glutathione systems and antioxidant defences. A full list of the pathways and mutants is given in Table 3. Compartmental *E*_GSH_ was subsequently estimated for all mutants with the experiment carried out in triplicate. For the calculation of *E*_GSH_ the pH values of the cytosol, mitochondrial matrix and peroxisome using targeted pHluorin probes were calculated at 7.5, 7.7 and 8 respectively and were used for *E*_GSH_ determination. Data obtained for cells in both exponential phase (A_600_ = ∼0.5) and stationary phase (A_600_ = 5–6) are presented in Dataset S2.

**Table 3:** Genes and known pathways important antioxidant function and resistance to oxidative stress [53].

Process	Genes involved
Transcription factors	*msn2, msn4, yap1, skn7 gnd1, gnd2, tal1,*
NADPH regeneration	*sol3, sol4,rpe1, tkl1, ald6, idp1, pos5, idp2, idp3*
GSH system	*glr1, gpx1, gp2, gpx3, grx1, grx2, grx3, grx4, grx5, grx6, grx7, grx8*
Thioredoxin system	*trx1, trx2, trx3, trr2*
Peroxiredoxins	*prx1, tsa1, tsa2, ahp1, dot5*
Antioxidants	*ctt1, cta1, sod1, sod2, ccp1*

Description and gene information were obtained from the *Saccharomyces Genome Database* (SGD).

**Figure 2:**
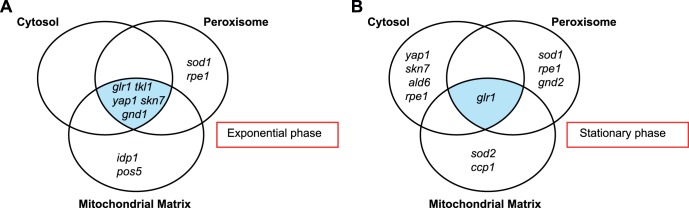
Functional overlap of mutants that have a more oxidizing cytosol and/or mitochondrial matrix and/or peroxisome. Mutants of known pathways involved in antioxidant defence and oxidative stress responses were analyzed in exponential (A_600_ = ∼0.5) and stationary phase (A_600_ = 5–6) in the cytosol, mitochondrial matrix and peroxisome. Analysis was carried out using flow cytometry and *E*_GSH_ estimated. 10,000 cells were counted in each conditions and each experiment was conducted in triplicate. Mutants with a Δ*E*GSH compared to wild type of 10 mV or more in A) exponential and B) stationary phase in each compartment tested are shown. Data taken Dataset S2.

From this targeted screen, genes affecting redox state in all organelles tested and genes unique for maintenance of redox state in a particular compartment were identified in both exponential and stationary phase. These are outlined in Figure 2A and 2B. Only 12 mutants (exponential phase) and 10 mutants (stationary phase) out of the 42 tested had altered redox in one or more of the compartments. Given the fact that a large number of genes and systems are known to deal with ROS and major perturbations of redox homeostasis, it is significant that mutations in most of these systems had no influence on redox state measured using roGFP2. This indicates that cellular redox homeostasis is tightly regulated and that only a subset of key oxidative stress proteins modulate *E*_GSH_.

The most prominent result was the requirement for glutathione reductase (Glr1p), NADPH regeneration systems and the transcription factors Yap1p and Skn7p for the maintenance of redox state across compartments, highlighting the fundamental role of these systems in redox homeostasis. The requirement for glutathione reductase was independent of growth phase unlike that of Yap1p and Skn7p, which appeared important only during exponential phase in all compartments. The identification of Yap1p and Skn7p as essential for cytosolic redox and response to stress indicate that both transcription factors sit at the boundary of maintenance-response systems. Basal levels of expression of the genes they regulate are important for steady-state redox homeostasis, while induction and activation of target genes appears important for mounting appropriate responses to oxidative stress.

From the data it appears that both the cytosol and mitochondrial matrix contain systems required to meet the unique redox and metabolic requirements of each organelle. The mitochondrial redox state was only affected by deletion of oxidative stress transcription factors and mitochondrial NADPH generating enzymes such as the NADH kinase encoded by *POS5* and the NADP^+^-dependent isocitrate dehydrogenase encoded by *IDP1* or mitochondrial ROS detoxifiers Mn-superoxide dismutase and cytochrome c peroxidase encoded by *SOD2* and *CCP1* respectively. The *ccp1* mutant had an altered matrix redox state in both growth phases, while *sod2* mutants only exhibited altered matrix redox in stationary phase, probably due to the increased requirement for Sod2p in stationary phase [27]. The significant role that these two genes play in maintaining mitochondrial redox state reflects the higher burden this compartment faces in terms of endogenous ROS production and subsequent detoxification as part of normal metabolism compared to other compartments. The effect of deleting *CCP1* is particularly interesting because in no other compartment did deletion of a peroxide-detoxifying gene result in alteration to redox state. Given that the mitochondrial matrix has other peroxide-detoxifying enzymes, for example Prx1p [28] and Cta1p [29], the data suggest a separate and significant role for Ccp1p in mitochondrial redox regulation.

In contrast, it was observed that peroxisomal redox was highly reliant on cytosolic rather than peroxisomal redox systems, with glutathione reductase activity, the operation of the pentose phosphate pathway and Cu,Zn superoxide dismutase all critical for maintenance of the redox environment in the peroxisome. Since very little is known about the redox systems in place in the peroxisome, the data may help to understand the manner in which the peroxisome regulates redox homeostasis and its relationship with other compartments. The dependence of the peroxisome on cytosolic pathways indicates a clear inter-relationship between peroxisomal and cytosolic redox homeostasis.

One outcome of this study has been the identification of a function for genes that are annotated in the *Saccharomyces Genome Database* as being “of unknown or putative function”. This has led to new or improved annotations of 10 genes (Table 4) on the basis of their involvement in redox homeostasis and potentially in oxidative stress responses. A good example is *HMS2,* which is a paralogue of *SKN7* arising from the ancient genome duplication [30]. *HMS2* is a putative transcription factor that is annotated by SGD as a “protein with similarity to heat shock transcription factors” involved in pseudohyphal growth under nitrogen depletion. Despite rapid protein divergence between *HMS2* and *SKN7*, *HMS2* shares the heat-shock factor and DNA-binding domains of Skn7p and has been predicted on the basis of microarray data to regulate a subset of responses to reducing conditions, heat shock and hydrogen peroxide [30]. Moreover, Hms2p is structurally very similar not only to Skn7p but also to Sfl1p, a transcriptional activator and repressor involved in the activation of several stress-response genes including Hsp30, which is strongly induced in response to various environmental stress conditions [31]. *HMS2* therefore, has a greater role in redox and oxidative stress regulation than previously thought, and may represent a transcriptional system acting in concert with *YAP1/SKN7* transcription factors to regulate redox state.

**Table 4:** Genes annotated as “putative or unknown function” identified as contributing to cytosolic redox homeostasis in this study and their oxidative stress phenotype(s).

Gene	Phenotype	Reference
*DOG2*	Induced by oxidative stress	[20]
*DGR2*	Implicated in stress response signalling	[54]
*RPI1*	Inhibitor of Ras-cAMP pathway	[55]
*PMP3*	Increased glutathione excretion	[24]
*HMS2*	Predicted to regulated some responses to reducing conditions, heat shock and hydrogen peroxide.Homolog of *SKN7*	[56]
*SVF1*	Inhibits reactive oxygen species generation and promotes cell survival under oxidative stress conditions	[57]
*ECM25*	Increased glutathione secretion; decreased oxidative stress resistance	[24], [58]
*ISF1*	Similar to Mbr1p, a protein involved in mitochondrial function and stress responses	[59]

## Discussion

The present study was undertaken with the aim of better understanding the genetic basis of the setting, sensing and maintenance of cellular redox homeostasis. The development of redox-sensitive fluorescent probes such as roGFP2 has enabled the high throughput study of redox state in an *in vivo* and real-time manner within distinct cellular compartments, circumventing many of the issues associated with traditional techniques for estimating redox state, such as mixing of compartmental glutathione pools and cell volume estimation. This study represents the first systematic genome-wide study analyzing cellular redox state and is the first to use genome-wide redox potential data to comprehensively understand the bases of redox homeostasis at a compartmental level. The combination of a genome-wide screen with a targeted sub-screen to identify factors contributing to sub-cellular redox homeostasis, highlights the value of *in vivo* redox probes in examining redox homeostasis and broader cellular issues involving redox perturbations. While roGFP2 predominantly equilibrates with the glutathione couple, its redox state also acts as a reflection of that of the protein-sulfhydryl redox buffering system. Protein-thiols contribute significantly to the cellular redox state [13] and measurements using roGFP2 are likely to reflect both the glutathione redox state and the protein-thiol network allowing the reporter to be a multi-dimensional read-out of redox conditions in the cell. Since the roGFP2 probe in the wild-type strain is in an almost fully reduced state across the three compartments studied, it could not be used to identify factors that resulted in more reducing cellular conditions. Re-engineered roGFP2 probes with various mid-point potentials [16], [32] could be used to study hyper-reducing conditions or investigate the redox state of the endoplasmic reticulum, which is too oxidizing to be studied using roGFP2. It must be kept in mind, however that there is not one definitive cellular redox state. Glutathione is present at a high concentration, which is an important feature of a buffer. Many other important redox couples exist in cells with varying interactions such as the NADP^+^/NADPH and Trx_OX_/Trx_RED_ couples. An increasing body of evidence is pointing to a more complex relationship between the glutathione and thioredoxin systems with a functional overlap between them. The thioredoxin system is important in preventing over-accumulation of GSSG in cells lacking glutathione reductase [33], [34]. Additionally, it has been found that the thioredoxin system is specifically involved in regulating glutathione redox potential in the nucleus even in the presence of glutathione reductase [35]. Future studies would be greatly enhanced by the use of a reporter constructs that equilibrate with the thioredoxin system and NADP^+^/NADPH couple to get a broader understanding of redox cellular dynamics.

From the data obtained, redox homeostasis in the cytosol, mitochondrial matrix and peroxisome is primarily controlled by regulated glutathione cycling in a growth-phase-independent manner since the *glr1* mutant exhibited the highest Δ*E*_GSH_. In exponential phase only the Yap1p and Skn7p transcription factors affected sub-cellular redox. The lack of redox shift in the *msn2* and *msn4* mutants indicates that while the Msn2p and MSn4p transcription factors are important for the general stress response [36], individually they play little or no role in maintenance of redox state. Therefore, it is unlikely that genes whose basal levels are highly regulated by Msn2p/Msn4p such as *GRX2* [37] would be required for steady state redox homeostasis and this is reflected in the results. The reduced need during stationary phase for Yap1p and Skn7p in all compartments is probably due to the fact that, during exponential phase, cells are actively growing and require the transcription of redox maintenance genes however this diminishes in stationary phase when active metabolism and protein synthesis [38] decline. In stationary phase, the need to regenerate existing reduced glutathione is more important and the activity of Glr1p is therefore predominant. Similarly, NADPH regeneration systems were important for maintaining the redox state in exponential phase with multiple NADPH regeneration mutants identified (cytosol - *tkl1*, *gnd1*; matrix – *tkl1, gnd1, idp1, pos5*; peroxisome - *rpe1, gnd1, tkl1*).

The finding that key oxidative stress response elements such as the transcription factors Yap1p and Skn7p, and GSH regeneration are essential for redox maintenance under steady state conditions alter the concept that systems involved in the maintenance of redox homeostasis and systems for response to ROS are distinct from each other. These data support the view that some oxidative stress response systems evolved from mechanisms that were important for maintenance of redox homeostasis. It is evident from this study that redox homeostasis is tightly regulated, with redundant systems in place to ensure the maintenance of the appropriate redox environment in each compartment. For the most part, several mutations would be needed to cause large shifts in the redox environment, highlighting the robustness of the cell in terms of redox control. The phenotype of single mutations does not always reflect the complete network of cell functions. We did not observe a significant redox shift in the *trx1* or *trx2* mutants. However, Drakulic and colleagues (2005) showed that the presence of either *TRX1* or *TRX2* was required for maintenance of *E*_GSH_ [12], despite the redundancy between cytosolic thioredoxins and glutaredoxins for cell viability [12]. In that study, the greatest shift in *E*_GSH_ from the wild-type in exponential phase was seen in the *trx1 trx2* double mutant. Therefore, while cellular redox systems are redundant and robust, complex redox relationships still do exist. Multiple mutations that have additive effects most likely represent pathways acting in concert for correct sensing and regulating of particular redox couples and redox homeostasis in general, for example the glutathione and thioredoxin systems. As state previously, there iare numerous connections between thioredoxin and glutathione systems. The thioredoxin reductase-glutathione reductase double mutant (*trr1 glr1*) is inviable [39] and the *trr1 glr1* double mutant is inviable [39] and only one single thioredoxin or glutaredoxin is essential for viability in *S. cerevisiae* [40]. Deletion of either or both thioredoxin reductases results in increased intracellular concentrations of oxidized and reduced glutathione [41], [42] and recently, it was found that the thioredoxin/thioredoxins reductase system could function as an alternative system to reduce oxidized glutathione *in vivo* in *S. cerevisiae* [33] and *Arabidopsis thaliana* [34].

There is a strong connection between mitochondrial function and redox homeostasis. Deletion of *CCP1* encoding cytochrome c peroxidase affected matrix redox state in both growth phases. As Ccp1p detoxifies hydrogen peroxide and other peroxides, it appears that detoxification of endogenous hydrogen peroxide is required to maintain mitochondrial matrix redox state. Interestingly, Ccp1p is also involved in oxidative stress signaling leading to activation of the Skn7p transcription factor [43]. This could explain the larger shift in matrix redox state observed in the *ccp1* mutant compared to the *skn7* mutant. Deletion of *CCP1* may simultaneously cause increased hydrogen peroxide accumulation and decreased activation of Skn7p, doubly affecting matrix redox state. The signaling function of Ccp1p provides a clear example of the existence of mitochondrial signaling molecules and of cross-compartmental redox signaling. Despite mitochondrial function being absent from those functions significantly enriched in the dataset using the GO and MIPS databases, it is clear that a relationship exists between some mitochondrial functions and maintenance of cytosolic redox state with many mutations affecting mitochondria identified as affecting cytosolic redox state. From previous work it is known that respiratory-incompetent cells have increased levels of GSSG in stationary phase relative to exponential phase and that mitochondrial function is required for resistance to oxidative stress [44]. Key mitochondrial proteins may act in the cellular signaling and sensing of redox change through retrograde redox signaling pathway(s). Since mitochondria are the largest site of endogenous ROS production, redox state changes in mitochondria may be an initial indicator for overall cellular redox dysfunction.

Little is known about peroxisomal redox despite the peroxisome being a significant site of endogenous ROS production. Despite this, the peroxisome exhibited distinctive redox characteristics, with a curious dependence on cytosolic proteins such as glutathione reductase (Glr1p), enzymes involved in the pentose phosphate pathway (Tkl1p, Rpe1p) and Cu, Zn superoxide dismutase (Sod1p) rather than peroxisomal proteins for the maintenance of its redox state in both exponential and stationary phase. These results raise some interesting possibilities regarding the nature of peroxisomal redox maintenance and its relationship with other compartments, the cytosol in particular. Free diffusion of small molecules between the cytosol and peroxisome has been hypothesized to account for the pool of GSH and GSSG and hence the redox state of the peroxisome [45] and the results are suggestive of peroxisomal redox state being reliant on cytosolic redox maintenance systems. However, recently, an isoform of glutathione reductase has been found to be dually targeted to the cytosol and peroxisome in *Arabidopsis thaliana* by a novel peroxisomal targeting sequence [46] raising the possibility that glutathione reductase may be localized to the peroxisome in other organisms and that the peroxisome may itself contain a discrete glutathione system.

This study identified approximately 100 genes involved in setting cytosolic redox state using the roGFP2 redox reporter construct. The processes identified highlight that certain redox-active systems traditionally thought of as stress response systems are critical for steady state redox homeostasis, and indicate that redox maintenance systems may have evolved to also be important for responding to reactive oxygen species. The study also found a phenotype of redox dysfunction in 12 genes of unknown or putative function and provides a rich source for further analysis of their cellular role and function in redox homeostasis.

## Materials and Methods

### Strains, Media and Growth Conditions

For genome-wide screening, the homozygous diploid knock-out mutant collection (Euroscarf) [15] (*MAT* ***a*** */MATα his3Δ1/his3Δ1 leu2Δ0/leu2Δ0 met15Δ0/MET15 LYS2/lysΔ0 ura3Δ0/ura3Δ0, KanMX4::*target gene) and the tetO_7_ promoter replacement collection (OpenBiosystems) [14] (*MAT* ***a*** *; his3Δ1; leu2Δ0; met15Δ0; URA::CMV-tTA,* kanR-tetO_7_-TATA::target gene) were used. Strains were grown in synthetic complete (SC) medium (0.17% yeast nitrogen base without amino acids, 0.5% ammonium sulfate, 2% w/v glucose) supplemented with appropriate amino acids and bases (Table S1). Dropout medium with the appropriate supplement omitted to provide plasmid selection is denoted by a subscript, for example SC medium lacking leucine is denoted as SC_LEU_. Media were solidified by the addition of 2% (w/v) agar. Strains were transformed with plasmids using a 96-well plate transformation protocol [47].

### Organelle-targeted roGFP2 Construction

Details of organellar-targeted roGFP2 constructs - pAG416-roGFP2 and pAG416-COX4roGFP2 are described in [18]. The peroxisome-targeted roGFP2 probe, pAG416-roGFP2-SKL was generated using PCR amplification and the Invitrogen Gateway System®. Briefly roGFP2 was PCR amplified from the plasmid pBinCM-TKTP-*GRX1*roGFP2 and the peroxisomal *C-*terminal targeting sequence seryl-lysyl-leucine (SKL) was added to the primer used for amplification. *attb1* or *attb2* sites were added to each primer as appropriate so that the resulting PCR products were flanked by a 5′ *attb1* site and a 3′ *attb2* site. The resulting PCR fragments were used for the Gateway^®^ BP clonase® reaction using the destination vector pDonr221. The pDonr221-roGFP2-SKL plasmid was generated using the Invitrogen Gateway^®^ system according to manufacturer’s instructions and the resulting plasmid pDonr221roGFP2-SKL was used for the Gateway LR^®^ clonase^®^ reaction using the destination vector pAG416GPD-ccdB [48]. pHluorin organelle-targeted probes were made in an identical manner to the roGFP2 based probes using pCB901YpHc as the starting vector encoding pHluorin a kind gift from R. Rao (Johns Hopkins University School of Medicine, Baltimore).

### Organelle-targeted roGFP2 Localization and Redox-sensitivity Verification

Correct localization of mitochondrial matrix-targeted roGFP2 was confirmed by confocal co-localization [18]. Correct localization of all plasmids used in the study was done via confocal microscopy (Figure S1). The redox responsiveness of cells expressing roGFP2-based plasmids was validated via flow cytometry and western blot. Cells were grown in in SC_URA_ for 48 h (30°C; 600 rpm) and inoculated into SC_URA_ (A_600_ = 0.001) and grown to exponential phase. Cells were treated with various concentrations of the strong reductant dithiothreitol and the oxidant diamide for 30 min and redox state of roGFP2 determined (data not shown).

### Analysis of Compartmental Redox Potential

Cells transformed with the roGFP2 constructs were grown in SC_URA_ for 2 d (30°C; 700 rpm), inoculated in SC_URA_ medium and grown with shaking at 25°C until appropriate cell yield had been reached. All analyses were carried out in 96-well plate format. For preliminary analyses, cytosolic redox state of the cells was analyzed in a fluorescent spectrophotometer (SpectraMax M2, Molecular Devices) with excitation at 405 nm and 488 nm. The emission after excitation at each wavelength was measured and then the R_405/488_ ratio calculated, with R_405/488_ representing the ratio of emission after excitation at 405 nm divided by the emission after excitation at 488 nm. Given that this was the first round of screening a lenient cut-off were used to identify mutants with altered cytosolic *E*_GSH_. In this preliminary screening we identified mutants that had any change in R_405/488_ and any change in either or both the emission after excitation at 405 nm and the emission after excitation at 488 nm. These were then further screened. Subsequent sub-screening and organellar redox analyses were conducted using a FACS Canto II flow cytometer (Becton Dickinson, Australia). For non-essential mutants, cells transformed with roGFP2 were grown to exponential phase (A_600_ 0.5–2.0) in SC_URA_. For screening of the tetO_7_ collection, cells transformed with roGFP2 (leucine selection) were grown in SC_LEU_ for 2 d (25°C; 700 rpm) and inoculated into SC_LEU_+10 µg/ml doxycycline and grown for 24 or 36 h (25°C; 700 rpm). Cells were then analyzed using the FACS Canto II (BD Bioscience) equipped with a laser that excites at 405 nm and 488 nm and emission was detected at 512 nm. The ratio of the emission after excitation at 405 and 488 nm was then calculated. The R_405/488_ and *E*_GSH_ calculated as previously reported [18], [49]. For each sample 10,000 cells were analyzed in at least three biological replicates. For analysis of significance differences in R_405/488_ and *E*_GSH_ values t-tests were conducted and all *E*_GSH_ values reported as significant were calculated using a *p-*value of 0.001. In each case the entire experiment was repeated at least twice. Data obtained were analyzed with FloJo version 7.4 (Tree Star Inc.).

### E_GSH_ Calculation

The following modified Nernst equation [49] was used to determine *E*_GSH_ from the roGFP2 405/488 ratio. Cells were also treated for 20 min with DTT (10 mM) or diamide (10 mM) to obtain measurements of the fully reduced or oxidized (respectively) forms of the roGFP2 probe that are necessary to calculate *E*_GSH_ using the following formulae derived from the Nernst equation.

Briefly, the degree of oxidation (*OxD*) of the roGFP2 sensor was calculated from
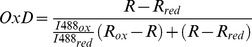
where *R* is the ratio of emission after excitation at 405/488 nm, *Rred* is the emission ratio of the fully reduced form following incubation with 10 mM DTT (20 min), *Rox* is the emission ratio of the fully oxidized form following incubation with 10 mM diamide (20 min) and *I*488ox and *I*488red are the respective emission intensities at 488 nm for the fully oxidized and fully reduced forms of a given sample. The redox potential was then estimated using:



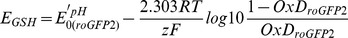
where *R* is the gas constant (8.315 J K^−1^ mol^−1^), *T* is the absolute temperature (298.15 K), *z* is the number of transferred electrons (2), *F* is the Faraday constant (9.648 10^4^ C mol^−1^) and *E*’^pH^_0(roGFP2)_ is the mid-point redox potential based on the standard mid-point potential *E*_0_ at 30°C and pH 7, adjusted for the estimated compartment pH and experimental temperature (20–25°C) according to:



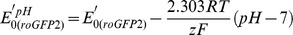
with the mid-point redox potential for roGFP2 (*E* ^0’^_roGFP2_) estimated as −272 mV.

For analysis of significance differences, t-tests were conducted on samples using α = 0.001.

### Analysis of Compartmental pH

Wild-type (BY4743) cells transformed with the pHluorin constructs were grown in SC_URA_ for 2 d (30°C; 700 rpm), inoculated in SC_URA_ medium and grown with shaking at 25°C until appropriate cell yield had been reached. Cells were analyzed using the FACS Canto II (BD Bioscience) with excitation at 405 nm and 488 nm and emission was detected at 512 nm. The ratio of the emission after excitation at 405 nm and 488 nm was then calculated and used for pH estimation. 10,000 cells were counted for each sample. Data obtained were analyzed with FlowJo version 7.4 software. To obtain pH values using the pHluorin probe in each compartment, a standard curve was established to allow estimation of intracellular pH by determining the fluorescence of digitonin-permeabilized cells incubated in buffers with pH values of 5 to 8 [50]–[52]. For calibration of the cytosolic-, mitochondrial matrix-, and peroxisomal-localized pHluorin, cells were suspended in 0.65 M sorbitol containing 100 mg/ml digitonin and incubated (10 min; 600 rpm; 24°C). Cells were pelleted, washed and resuspended in calibration buffer containing 50 mM 2-(*N*-morpholino)ethanesulfonic acid (MES), 50 mM *N*-(2-hydroxyethyl)piperazine-Ń-(2-ethanesulfonic acid) (HEPES), 50 mM KCl, 50 mM NaCl, 200 mM ammonium acetate, 10 mM NaN_3_, 10 mM 2-deoxyglucose, 75 µM monensin and 10 µM nigericin, titrated to different pH values using NaOH within the range of pH 6.0–8.0. A calibration curve was plotted using the ratio of fluorescence intensity at 405 nm and 488 nm (after background subtraction) against pH.

### Analysis of Genome-wide Data

Cellular processes over-represented in the genome-wide screen data were identified using the Gene Ontology (GO) and Munich Information Centre for Protein Sequences (MIPS) databases via FunSpec [19] with the *p*-value threshold set at 0.01. FunSpec calculated *p*-values using a hypergeometric distribution, and the *p*-value represents the probability that the intersection of a given list with any given functional category occurs by chance. The *p*-values were Boneferroni corrected to account for spurious significance due to multiple testing over the categories of a database.

## Supporting Information

Figure S1 **Confocal microscopy images of yeast cells expressing peroxisomal roGFP2.**
(EPS)

Figure S2 **Verification of organelle-targeted roGFP2 responsiveness to redox state.**
(EPS)

Table S1 **Supplements for synthetic complete (SC) medium.**
(DOC)

Dataset S1 **Non-essential mutants identified as having a significantly more oxidized cytosol than wild type.**
(XLS)

Dataset S2 ***E*** **_GSH_ in the cytosol, mitochondrial matrix and peroxisome of cells lacking genes involved in redox homeostasis and antioxidant defence in exponential and stationary phase.**
(DOC)
