# Supplementary material for: A Genome-Wide Screen in Yeast Identifies Specific Oxidative Stress Genes Required for the Maintenance of Sub-Cellular Redox Homeostasis
Source: PLoS One. 2012 Sep 6;7(9):e44278. doi: 10.1371/journal.pone.0044278 (PMC3435413; doi:10.1371/journal.pone.0044278)
Supplement: Table S1 — Supplements for synthetic complete (SC) medium. (DOC) [file pone.0044278.s003.doc]

Supplementary Table S1: Supplements for synthetic complete (SC) medium

| **Supplement** | **Medium Concentration**  **(mg per l)** | **Supplement** | **Medium Concentration**  **(mg per l)** |
| --- | --- | --- | --- |
| L- Adenine | 18 | L- Leucine | 260 |
| L- Alanine | 76 | L-Lysine | 76 |
| L- Arginine | 76 | L- Methionine | 76 |
| L- Asparagine | 76 | L- Phenylalanine | 76 |
| L- Aspartic acid | 76 | L- Proline | 76 |
| L- Cysteine | 76 | L- Serine | 76 |
| L- Glutamic acid | 76 | L- Threonine | 76 |
| L- Glutamine | 76 | L- Tryptophan | 76 |
| Glycine | 76 | L- Tyrosine | 76 |
| L- Histidine | 211 | L-Valine | 76 |
| L- Isoleucine | 76 | Uracil | 22.5 |
