# Supplementary material for: A Genome-Wide Screen in Yeast Identifies Specific Oxidative Stress Genes Required for the Maintenance of Sub-Cellular Redox Homeostasis
Source: PLoS One. 2012 Sep 6;7(9):e44278. doi: 10.1371/journal.pone.0044278 (PMC3435413; doi:10.1371/journal.pone.0044278)
Supplement: Dataset S2 — E GSH in the cytosol, mitochondrial matrix and peroxisome of cells lacking genes involved in redox homeostasis and antioxidant defence in exponential and stationary phase. (DOC) [file pone.0044278.s005.doc]

**Dataset S2: *E*_GSH_ in the cytosol, mitochondrial matrix and peroxisome of cells lacking genes involved in redox homeostasis and antioxidant defence in exponential and stationary phase.** Each mutant was separately transformed with the compartmental specific roGFP2 constructs. Transformed cells were grown to exponential phase (A_600_ = ~0.5) and stationary phase (A_600_ = 5-6) and *E*_GSH_ estimated by flow cytometry analysis as described in the Materials and Methods. 10,000 cells were analyzed and data are the mean of triplicates ± standard deviation. EGSH values that were significantly different to wild-type are indicated in red bold font.

|  |  |  | **CYTOSOL** | |  | **MITOCHONDRIAL MATRIX** | |  | **PEROXISOME** | |  |
| --- | --- | --- | --- | --- | --- | --- | --- | --- | --- | --- | --- |
| **Functional Group** |  | **Description** | **Exponential Phase**  ***E*_GSH_ ± SD**  **(-mV)** | **Stationary Phase**  ***E*_GSH_ ± SD**  **(-mV)** | | **Exponential Phase**  ***E*_GSH_ ± SD**  **(-mV)** | **Stationary Phase**  ***E*_GSH_ ± SD**  **(-mV)** | | **Exponential Phase**  ***E*_GSH_ ± SD**  **(-mV)** | **Stationary Phase**  ***E*_GSH_ ± SD**  **(-mV)** | |
|  | WT |  | 349 ± 3 | 335 ± 3 | | 353 ± 3 | 348 ± 3 | | 346 ± 4 | 340 ± 4 | |
| Transcription Factors |  |  |  |  | |  |  | |  |  | |
|  | *yap1* | Basic leucine zipper (bZIP) transcription factor | **320 ± 4** | **322 ± 3** | | **337 ± 4** | 348 ± 3 | | **336 ± 4** | 333 ± 4 | |
|  | *skn7* | Nuclear response regulator and transcription factor | **319 ± 5** | **320 ± 3** | | **340 ± 6** | 341 ± 3 | | **337 ± 5** | 332 ± 5 | |
|  | *msn2* | Transcriptional activator related to Msn4p | 345 ± 3 | 327 ± 4 | | 343 ± 4 | 346 ± 4 | | 340 ± 4 | 333 ± 4 | |
|  | *msn4* | Transcriptional activator related to Msn2p | 347 ± 3 | 328 ± 3 | | 345 ± 4 | 348 ± 3 | | 343 ± 3 | 332 ± 4 | |
| NADPH regeneration |  |  |  |  | |  |  | |  |  | |
|  | *gnd1* | 6-phosphogluconate dehydrogenase | **320± 5** | 335±5 | | 314± 5 | 349± 4 | | **343± 3** | 330± 4 | |
|  | *gnd2* | 6-phosphogluconate dehydrogenase | 335± 4 | 333±6 | | 355± 3 | 340± 5 | | 339± 5 | **325± 5** | |
|  | *sol3* | 6-phosphogluconolactonase | 343± 5 | 340±5 | | 350± 6 | 345± 3 | | 345± 6 | 338± 4 | |
|  | *sol4* | 6-phosphogluconolactonase | 339± 6 | 329±4 | | 349± 3 | 344± 3 | | 344± 4 | 335± 5 | |
|  | *rpe1* | D-ribulose-5-phosphate 3-epimerase | 345± 4 | **320±5** | | **338± 5** | 339± 4 | | **349± 3** | **329± 6** | |
|  | *tal1* | Transaldolase | 339 ± 5 | 332 ± 4 | | 350 ± 6 | 314 ± 4 | | 347 ± 4 | 337 ± 3 | |
|  | *tkl1* | Transketolase; similar to *TKL2* | **330 ± 4** | 329 ± 3 | | **336 ± 3** | 319 ± 5 | | **335 ± 6** | 337 ± 4 | |
|  | *idp1* | Mitochondrial NADP^+^-specific isocitrate dehydrogenase | 345 ± 3 | 327 ± 4 | | **321 ± 4** | 310 ± 4 | | 341 ± 4 | 332 ± 3 | |
|  | *idp2* | Cytosolic NADP^+^-specific isocitrate dehydrogenase | 349 ± 4 | 329 ± 4 | | 343 ± 3 | 349 ± 4 | | 346 ± 4 | 339 ± 3 | |
|  | *idp3* | Peroxisomal NADP^+^-dependent isocitrate dehydrogenase | 338 ± 4 | 327 ± 3 | | 345 ± 4 | 347 ± 3 | | 343 ± 3 | 335 ± 4 | |
|  | *pos5* | Mitochondrial NADH kinase | 337 ± 7 | 325 ± 5 | | **319 ± 6** | 315 ± 4 | | 347 ± 4 | 332 ± 3 | |
|  | *ald6* | Cytosolic aldehyde dehydrogenase, | 334 ± 3 | **320 ± 3** | | 331 ± 3 | 342 ± 3 | | 323 ± 3 | 331 ± 3 | |
| Glutathione system |  |  |  |  | |  |  | |  |  | |
|  | *glr1* | Cytosolic and mitochondrial glutathione oxidoreductase, | **275 ± 4** | **272 ± 5** | | **272± 4** | **258 ± 5** | | **276± 5** | **273 ± 4** | |
|  | *gpx1* | Phospholipid hydroperoxide glutathione peroxidase | 354 ± 5 | 327 ± 3 | | 350 ± 3 | 341 ± 3 | | 347 ± 3 | 338 ± 3 | |
|  | *gpx2* | Phospholipid hydroperoxide glutathione peroxidase | 344 ± 3 | 333 ± 2 | | 343 ± 5 | 344 ± 3 | | 341 ± 4 | 333 ± 4 | |
|  | *gpx3* | Phospholipid hydroperoxide glutathione peroxidase | 344 ± 3 | 329 ± 3 | | 342 ± 3 | 343 ± 4 | | 339 ± 4 | 323 ± 4 | |
|  | *grx1* | Cytoplasmic di-thiol glutaredoxins | 354 ± 5 | 337 ± 3 | | 350 ± 4 | 344 ± 4 | | 347 ± 3 | 340 ± 3 | |
|  | *grx2* | Cytoplasmic di-thiol glutaredoxins | 359 ± 4 | 335 ± 3 | | 344 ± 4 | 357 ± 3 | | 347 ± 4 | 349 ± 3 | |
|  | *grx3* | Nuclear shuttling monothiol glutaredoxins | 344 ± 3 | 331 ± 4 | | 347 ± 6 | 353 ± 3 | | 345 ± 4 | 346 ± 4 | |
|  | *grx4* | Nuclear shuttling monothiol glutaredoxins | 343 ± 3 | 335 ± 4 | | 347 ± 3 | 355 ± 4 | | 344 ± 3 | 348 ± 3 | |
| Thioredoxin system |  |  |  |  | |  |  | |  |  | |
|  | *trr2* | Mitochondrial thioredoxin reductases | 354 ± 5 | 331 ± 3 | | 346 ± 5 | 346 ± 3 | | 344 ± 3 | 342 ± 6 | |
|  | *trx1* | Cytoplasmic thioredoxin | 352 ± 4 | 337 ± 3 | | 342 ± 4 | 343 ± 3 | | 347 ± 3 | 334 ± 3 | |
|  | *trx2* | Cytoplasmic thioredoxin | 350 ± 5 | 333 ± 4 | | 350 ± 5 | 352 ± 4 | | 348 ± 5 | 335 ± 4 | |
|  | *trx3* | Mitochondrial thioredoxin | 352 ± 3 | 332 ± 3 | | 348 ± 3 | 347 ± 3 | | 343 ± 3 | 339 ± 3 | |
|  |  |  |  |  | |  |  | |  |  | |
| Peroxiredoxins | *tsa1* | Cytoplasmic thioredoxin peroxiredoxin | 347 ± 3 | 327 ± 3 | | 343 ± 3 | 342 ± 5 | | 342 ± 3 | 348 ± 5 | |
|  | *tsa2* | Cytoplasmic thioredoxin peroxiredoxin | 351 ± 4 | 333 ± 5 | | 352 ± 4 | 343 ± 3 | | 348 ± 4 | 347 ± 4 | |
|  | *ahp1* | Cytoplasmic thioredoxin peroxiredoxin | 352 ± 4 | 329 ± 4 | | 350 ± 4 | 345 ± 4 | | 346 ± 4 | 342 ± 6 | |
|  | *dot5* | Nuclear thiol peroxidase | 348 ± 3 | 335 ± 3 | | 342 ± 5 | 352 ± 4 | | 343 ± 3 | 337 ± 4 | |
|  | *prx1* | Mitochondrial peroxiredoxin | 347± 4 | 330± 5 | | 339± 3 | 342± 4 | | 345 ± 5 | 340± 6 | |
| Antioxidant function |  |  |  |  | |  |  | |  |  | |
|  | *ctt1* | Cytosolic catalase T | 350 ± 4 | 322 ± 4 | | 350 ± 4 | 346 ± 3 | | 348 ± 5 | 342 ± 6 | |
|  | *cta1* | Peroxisomal catalase A | 349 ± 4 | 329 ± 3 | | 350 ± 4 | 348 ± 4 | | 348 ± 3 | 344 ± 6 | |
|  | *sod1* | Cytosolic copper-zinc superoxide dismutase | 333 ± 6 | 334 ± 3 | | 345 ± 6 | 342 ± 3 | | **316 ± 4** | **312 ± 3** | |
|  | *sod2* | Mitochondrial matrix manganese superoxide dismutase | 346± 5 | 332± 5 | | 340± 4 | **328± 5** | | 347± 5 | 337± 6 | |
|  | *ccp1* | Mitochondrial cytochrome-c peroxidase | 342± 5 | 335± 6 | | **326± 6** | **338± 4** | | 345± 4 | 334± 4 | |
